# Evaluation of relevant indicators in the diagnosis of cervical lymph node metastasis in papillary thyroid cancer

**DOI:** 10.3389/fonc.2026.1859596

**Published:** 2026-06-29

**Authors:** Jun Hu, Wenjia Shen, Jiawei Feng, Yong Jiang

**Affiliations:** 1 Department of Thyroid Surgery, The Third Affiliated Hospital of Soochow University, Changzhou First People’s Hospital, Changzhou, Jiangsu, China; 2 Department of Ultrasound, The Third Affiliated Hospital of Soochow University, Changzhou First People’s Hospital, Changzhou, Jiangsu, China

**Keywords:** cervical lymph node, papillary thyroid carcinoma, the ratio of serum Tg to FNA-Tg, thyroglobulin, thyroglobulin washout of fine needle aspiration, ultrasound-guided fine-needle aspiration cytology

## Abstract

**Background:**

This study aims to investigate the role of FNAC, FNA-Tg, the ratio of FNA-Tg to serum Tg, and their combined application in evaluating cervical lymph node metastasis in papillary thyroid cancer (PTC) to identify a more effective diagnostic strategy.

**Methods:**

A total of 284 patients with 319 cervical lymph node (LN) samples enrolled from January 2022 to March 2024 were retrospectively analyzed. All patients underwent thyroid surgery and neck lymph node dissection. Relevant indicators of thyroid status were collected from patients.

**Results:**

Serum Tg levels were associated with FNA-Tg levels. Compared with FNA-Tg alone, the ratio of FNA-Tg to serum Tg also played an important role in the diagnosis of lymph node metastasis. Moreover, when FNA-Tg or the ratio was combined with FNAC, diagnostic sensitivity, specificity, accuracy, and positive predictive value for detecting metastatic PTC lymph nodes were significantly higher, with statistically significant differences.

**Conclusion:**

Both FNA-Tg levels and the ratio of FNA-Tg to serum Tg can enhance the diagnostic efficacy of FNAC for cervical LN metastasis in PTC and demonstrate high diagnostic value. They are superior to FNAC alone. When combined with FNAC, they can further improve diagnostic efficiency. A higher positive rate in preoperative cervical lymph node diagnosis can not only provide a stable basis for selecting the subsequent surgical approach but also play an important role in pathological staging and prognostic follow-up.

## 1 Introduction

Thyroid cancer, with an annual growth rate of 4.5%–6.6%, has become one of the most common endocrine malignancies worldwide (1). Papillary thyroid carcinoma (PTC) accounts for 75%–85% of all thyroid malignancies and has shown a significant upward trend in recent years (2, 3). The prognosis of PTC is relatively better than that of other types of thyroid cancer, and patients have a long survival period. However, cervical lymph node metastasis is detected in 20%–60% of thyroid cancer patients at initial diagnosis or during follow-up because of recurrence (4, 5). Given that the presence of cervical lymph node metastasis is a crucial factor influencing radical neck dissection, particularly the extent of surgical resection, timely and precise evaluation of lymph node metastasis is of great significance for the treatment of patients with PTC.

Physical examination and ultrasound are the preferred methods for evaluating suspected thyroid nodules and cervical lymph nodes. Suspicious lymph nodes should be further examined by fine needle aspiration cytology (FNAC) (6, 7), which can effectively differentiate benign from malignant cervical lymph nodes. However, the diagnostic efficacy of FNAC is influenced by the operator’s experience, sample cellularity, and blood contamination, with a non-diagnostic rate close to 20%–30% (8, 9). It may have an enormous impact on the diagnosis and treatment of thyroid cancer, the choice of surgical approach, and postoperative follow-up in patients with PTC.

Recent studies have shown that the measurement of thyroglobulin (Tg) from FNA washout fluid (FNA-Tg) is beneficial for detecting cervical lymph node (LN) metastasis (10, 11) and serves as an effective supplement to FNAC. Elevated levels of FNA-Tg in lymph nodes may indicate differentiated thyroid cancer with cervical LN metastasis. Due to its high sensitivity and specificity in the diagnosis of cervical LN metastasis, FNA-Tg is now recommended for routine clinical use (12, 13). Therefore, the combination of FNA-Tg and FNAC has substantial diagnostic value for patients with PTC and cervical LN metastasis. Nevertheless, several challenges remain. The lack of a standardized and unified diagnostic cutoff value for FNA-Tg is the most critical problem in distinguishing benign from malignant cervical lymph nodes. The optimal cutoff values vary widely across clinical centers (14). Moreover, the detection of FNA-Tg may still be influenced by multiple factors, including serum Tg (sTg), serum thyroglobulin antibody (sTgAb), serum thyroid-stimulating hormone (TSH) levels, and thyroid status (15, 16).

Therefore, this study aims to analyze the factors that may influence FNA-Tg levels and to determine whether better indicators exist for diagnosing cervical lymph node metastasis. Furthermore, we systematically analyzed and summarized the diagnostic value of FNAC, FNA-Tg, and their combined application for detecting cervical lymph node metastasis in patients with PTC. Notably, the ratio of FNA-Tg to serum Tg (FNA-Tg/sTg) was explored as a novel evaluation indicator to enhance diagnostic sensitivity and specificity for cervical LN metastasis.

## 2 Materials and methods

### 2.1 Patients and study design

This retrospective cohort study received ethical approval from the Institutional Review Board of Changzhou First People’s Hospital. All participants agreed to have their clinical records used in this study and provided written informed consent. From our department’s prospective surgical database, we retrospectively reviewed 319 lymph node samples from 284 consecutive patients who underwent thyroidectomy at Changzhou First People’s Hospital between January 2022 and December 2024 and were pathologically diagnosed with thyroid cancer accompanied by suspicious cervical lymph node metastasis. All selected patients underwent ultrasound assessment of thyroid status, relevant thyroid function testing, fine-needle aspiration of lymph nodes, and FNA-Tg examination. In this study, the eligibility criteria were as follows: (1) all patients underwent surgery after a definite diagnosis; (2) the primary lesion was located in the thyroid gland; (3) postoperative pathology confirmed PTC; (4) no history of other malignant tumors; (5) no family history of thyroid cancer; and (6) complete clinical data. The exclusion criteria were as follows: (1) non-PTC cancers (medullary/follicular/anaplastic) or mixed-type thyroid cancer; (2) a history of cervical lymph node tuberculosis or other cervical tumors; (3) a history of neck radiation or familial cancer syndrome; (4) a history of receiving radiotherapy, chemotherapy, ^131^I ablation, or other tumor-related treatments before lymph node dissection; and (5) incomplete clinical data or missing follow-up.

### 2.2 Ultrasound and fine needle aspiration

Ultrasound is currently the most common examination method for the initial evaluation of suspected metastatic cervical lymph nodes (LNs) in papillary thyroid carcinoma (PTC) (17). However, the diagnostic accuracy is greatly influenced by the skill and experience of the ultrasound operator. For suspicious LNs, fine-needle aspiration (FNA) is recommended for further evaluation. Due to the limited detection rate of FNA for cervical LNs, false-negative results may occur. Therefore, FNA-Tg has been adopted as a powerful adjunctive tool for diagnosing cervical LN metastasis. Accordingly, to mitigate the impact of relevant confounding factors, the ratio of FNA-Tg to serum Tg (FNA-Tg/sTg) was introduced as a novel diagnostic indicator.

Firstly, the ultrasonographic characteristics of suspected cervical lymph nodes (LNs) in patients with PTC require preliminary evaluation using B-mode ultrasound, including: (1) obvious morphological changes (round or irregular shape, with a longitudinal-to-transverse diameter ratio <1.5); (2) unclear boundary between the cortex and medulla; (3) increased or heterogeneous echogenicity; (4) lymph node fusion; (5) disappearance of the lymphatic hilum; and (6) punctate or microcalcifications. Subsequently, FNA and FNA-Tg were performed to verify these ultrasonographic findings. This procedure utilized a 21–25-gauge needle without suction. At least two needle passes were performed for each nodule. Under real-time ultrasound guidance, the needle was inserted into the suspected lymph node and moved back and forth until sufficient aspirate material was collected in the needle hub. The smeared material on glass slides was then immediately fixed with 95% ethanol for Papanicolaou staining. The sampling needle was rinsed with 1.0 mL of normal saline, and the resulting solution was sent for FNA-Tg measurement (18).

### 2.3 Measurement of sTg, TSH, and TgAb

Three to five venous blood samples were collected from each patient for measurement using an automated electrochemiluminescence immunoassay. The concentration detection range for both sTg and FNA-Tg was 0.04 ng/mL–500.00 ng/mL. When the concentration exceeded 500.00 ng/mL, the value was recorded as 500.00 ng/L during data processing. When the concentration was below 0.04 ng/mL, the value was recorded as 0.04 ng/mL. Meanwhile, serum levels of thyroid-stimulating hormone (TSH) and anti-thyroglobulin antibody (TgAb) were also measured using a chemiluminescent microparticle immunoassay.

### 2.4 Marking for punctured lymph nodes

A fine needle was used to inject approximately 0.1 mL–0.2 mL of nano-carbon suspension into the targeted lymph node after the suspicious lymph node was localized by ultrasound. Simultaneously, the size and location of the lymph node were recorded in detail to facilitate accurate identification during surgical resection.

### 2.5 Diagnosis of benign and metastatic lymph nodes and surgical plan

**Figure 1:**
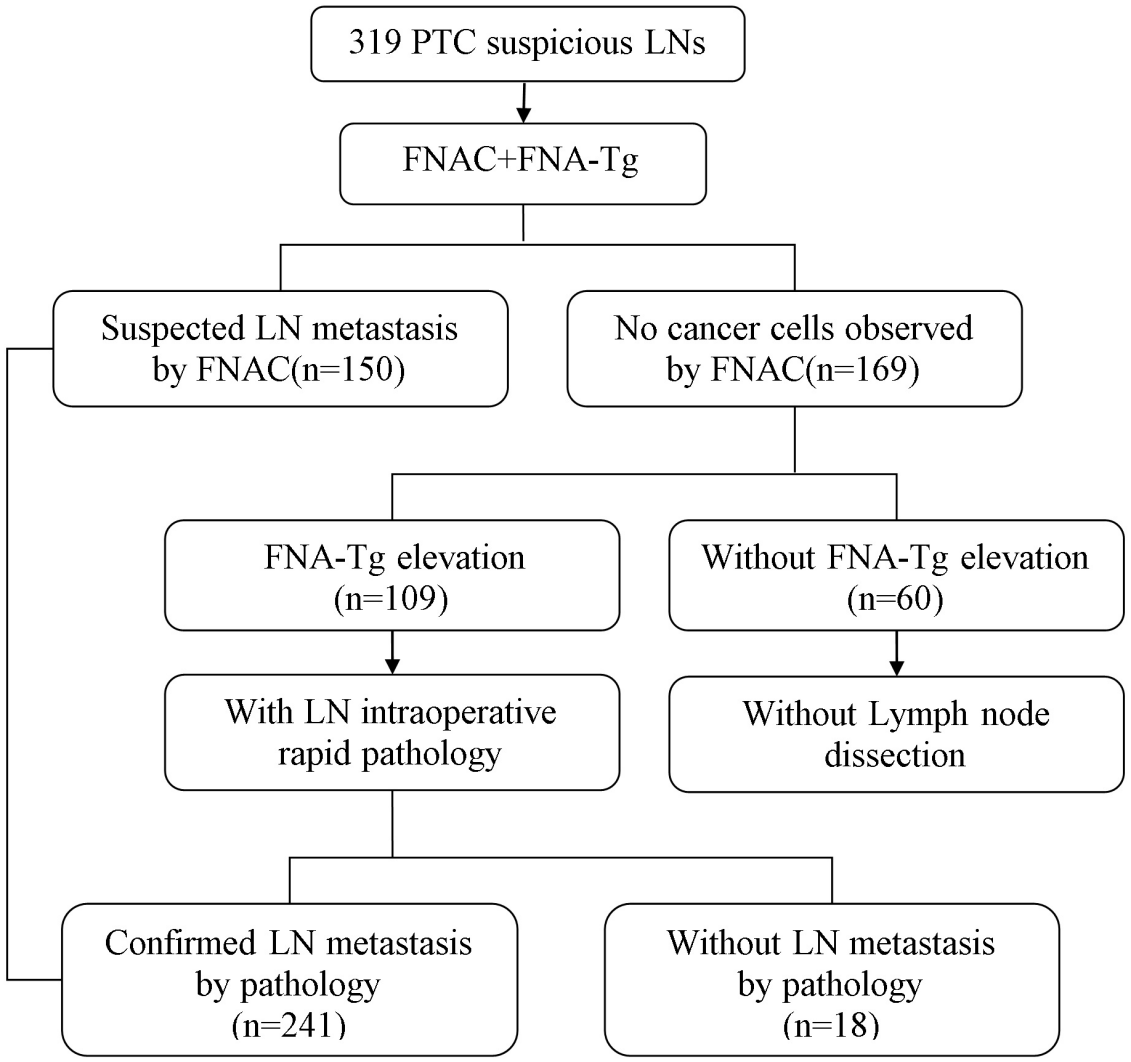
Enrollment flow chart of the evaluated patients and lymph nodes. FNAC, Fine-needle aspiration cytology; Tg, Thyroglobulin; FNA-Tg, Thyroglobulin from FNA washout fluid; LN, lymph node.

Based on clinical guidelines and practical experience, when the fine-needle aspiration (FNA) results of cervical lymph nodes were negative and the FNA-Tg level was below the normal cutoff, the lymph node was identified as benign, and no additional lymph node dissection was required. When the FNA results indicated malignancy, regional lymph node dissection was performed. When the FNA results were negative but the FNA-Tg level was elevated, intraoperative rapid pathological examination of the marked cervical lymph nodes was performed before deciding the next treatment step (**Figure 1**). Ultimately, histopathological results served as the gold standard for diagnosing cervical lymph node metastasis; otherwise, lymph nodes were considered non-metastatic.

### 2.6 Statistical analysis

Continuous variables were presented as median (lower quartile, upper quartile) and compared between groups using the Mann–Whitney U test. Categorical variables were presented as count (proportion) and compared using the chi-square test. Linear regression analysis was performed to explore the relationship between multiple factors and the dependent variable. ROC curve analysis was conducted, and Youden’s index was calculated to determine the optimal cutoff value. Sensitivity, specificity, positive predictive value, negative predictive value, accuracy, receiver operating characteristic (ROC) curve analysis, and area under the curve (AUC) were used to evaluate the diagnostic performance of FNAC and FNA-Tg. A P-value <0.05 was considered statistically significant. All statistical analyses were performed using SPSS version 25.0 (IBM Corp., Armonk, NY, USA).

## 3 Results

### 3.1 Comparison of clinicopathological characteristics of PTC between metastatic cervical lymph nodes and benign lymph nodes

A total of 319 lymph node (LN) samples from 284 patients (88 males and 196 females) were collected in this study. The median age of the metastatic group and the non-metastatic group was 56 years (45, 70) and 48 years (44, 63), respectively. Among the 319 LN specimens, 150 LNs were suspected of having metastatic potential based on FNAC results and subsequently underwent cervical lymph node dissection. Of these, 147 cases were histopathologically confirmed as metastatic. Among the remaining 169 specimens with negative FNAC results, 60 had FNA-Tg levels below the normal cutoff and did not require additional regional lymph node dissection. For the 109 specimens with normal or elevated FNA-Tg levels, intraoperative rapid pathological examination was performed to determine the next surgical approach. Among these, 18 were confirmed to have no lymph node metastasis, whereas 91 underwent metastatic lymph node dissection (**Figure 1**).

**Figure 2:**
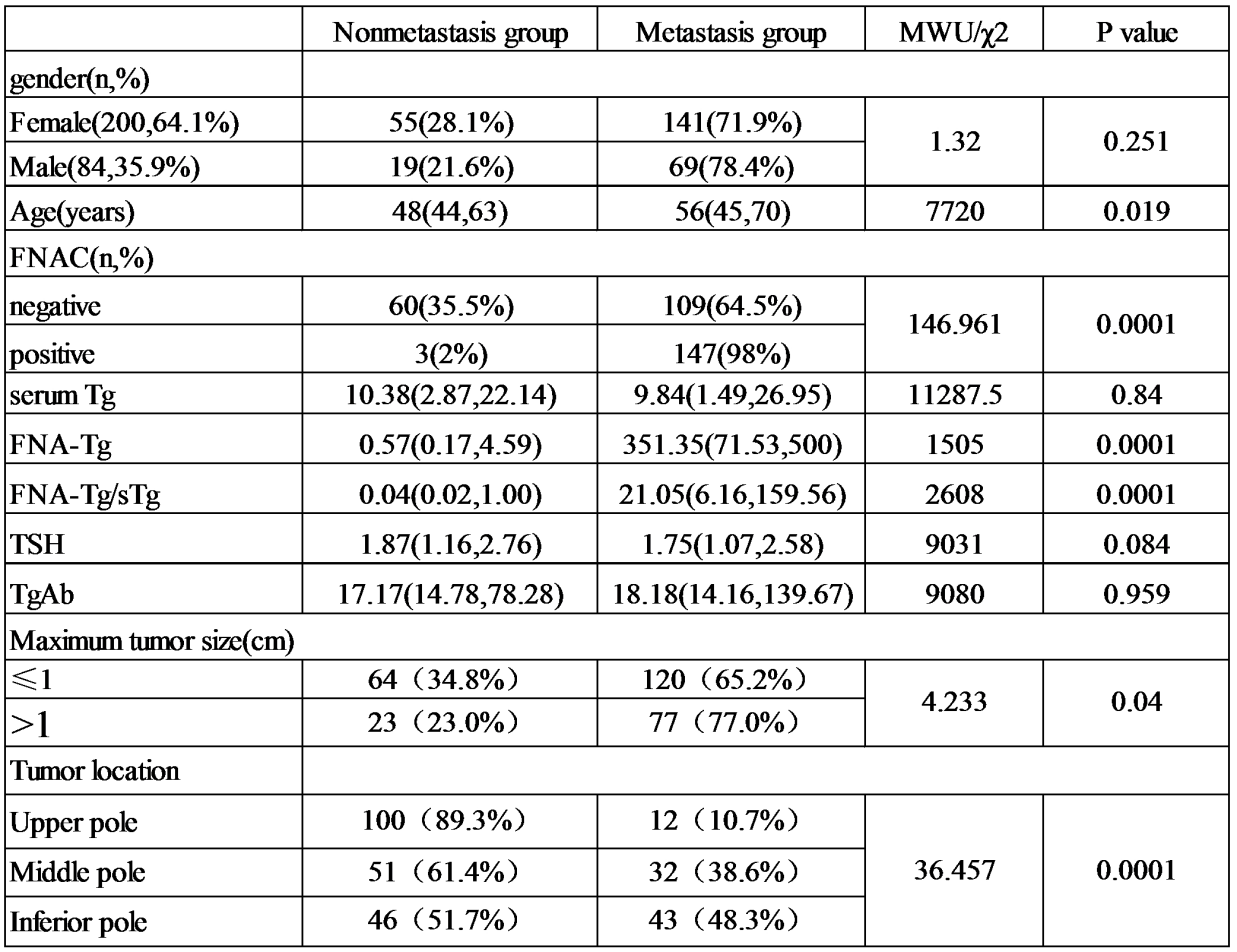
Clinical characteristics of 284 patients and 319 lymph nodes. FNAC, Fine-needle aspiration cytology; FNA-Tg, Thyroglobulin from FNA washout fluid; LN, lymph node; TSH, Thyroid stimulating hormone; TgAb, thyroglobulin antibody. Continuous variables were analyzed using the “Mann–Whitney U Test” (MWU) and presented as “median (lower quartile, upper quartile)”. Categorical variables were analyzed using the “Chi-Squared Test” and presented as “count (proportion).”.

No significant difference was observed between groups in serum Tg, TgAb, and TSH levels. There were statistically significant differences between the two groups in FNAC, FNA-Tg, and FNA-Tg/sTg (all P <0.05). In addition, patients in the metastatic group had larger maximum tumor size and more distinct tumor location characteristics compared with those in the non-metastatic group (all P <0.05; **Figure 2**).

### 3.2 Relevance between FNA-Tg and other factors

**Figure 3:**
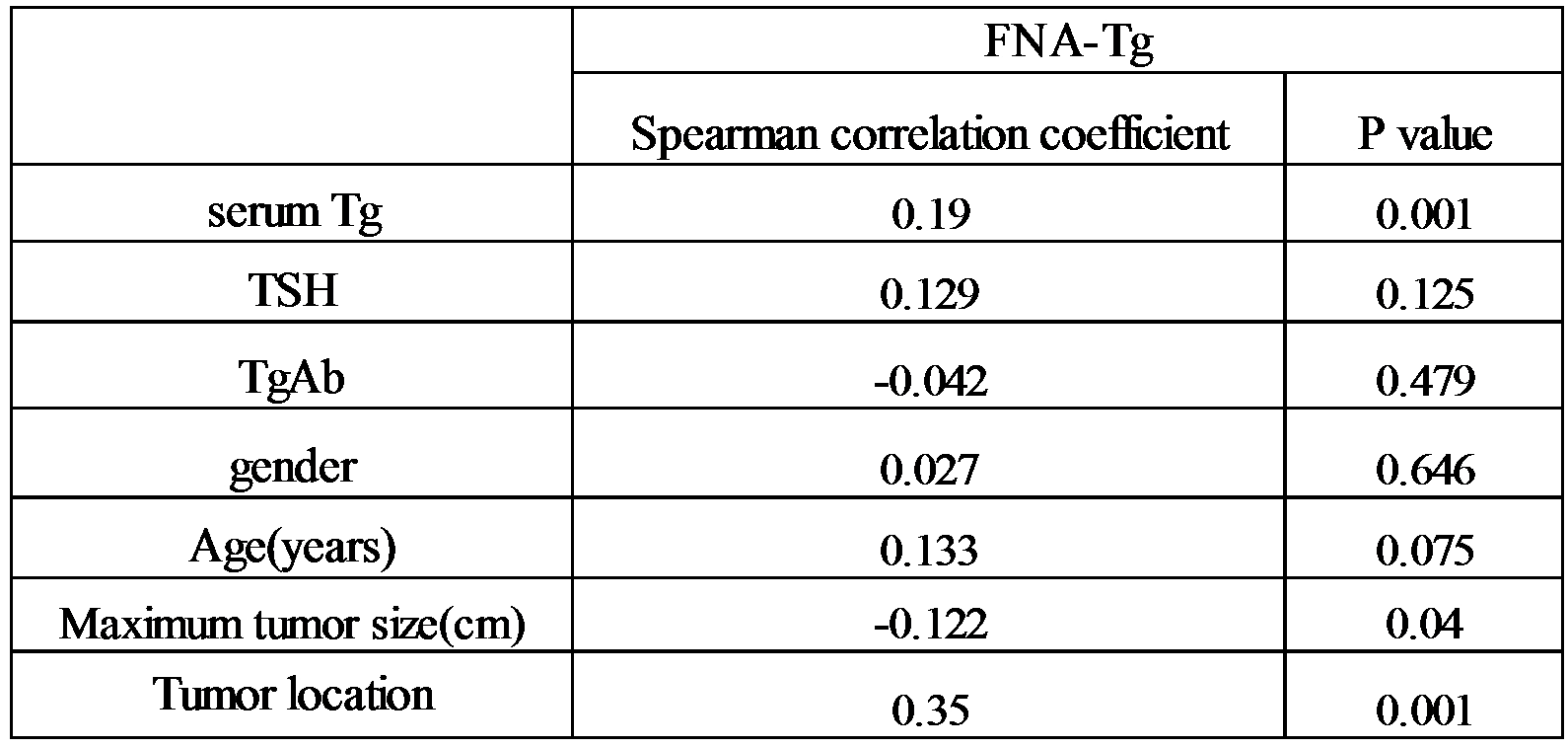
Spearman correlation analysis of FNA-Tg with clinical factors. Tg, Thyroglobulin; FNA-Tg, Thyroglobulin from FNA washout fluid; TSH, Thyroid stimulating hormone; TgAb, thyroglobulin antibody.

Spearman correlation analysis was performed to investigate the factors influencing FNA-Tg levels in metastatic cervical lymph nodes. The dependent variable was FNA-Tg, and the independent variables included serum Tg, TgAb, TSH, sex, age, maximum tumor size, and tumor location. The correlation coefficient between serum Tg and FNA-Tg was 0.19, indicating a weak positive correlation, which was statistically significant (P = 0.001). The correlation coefficient between maximum tumor size and FNA-Tg was −0.122, indicating a weak negative correlation, which was statistically significant (P = 0.04). The correlation coefficient between tumor location and FNA-Tg was 0.35, indicating a moderate positive correlation, which was statistically significant (P = 0.04). TgAb, TSH, age, and sex showed no statistically significant correlation with FNA-Tg (**Figure 3**).

### 3.3 ROC analysis for cutoff values of FNA-Tg and the ratio of FNA-Tg to serum Tg

**Figure 4:**
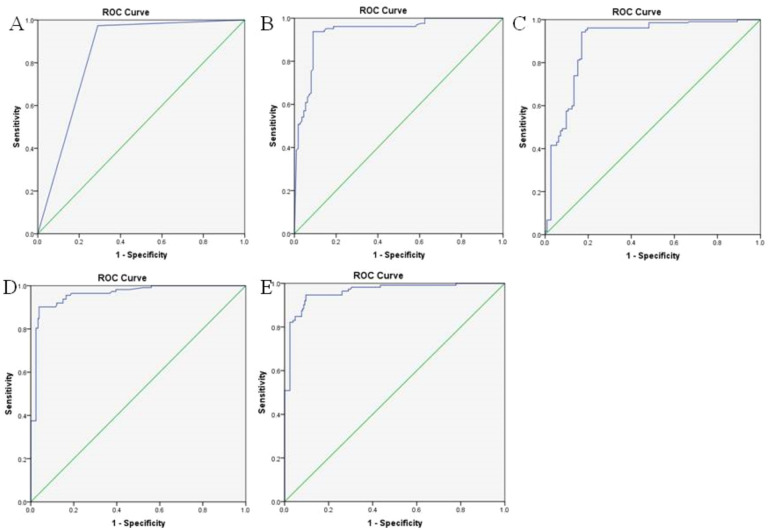
ROC curve of relevant methods for diagnosing cervical lymph nodes in PTC patients. **(A)** ROC curve of FNAC (AUG: 0.842, P <0.0001). **(B)** ROC curve of FNA-Tg (AUG: 0.937, P <0.0001). **(C)** ROC curve of the ratio of FNA-Tg to Tg (AUG: 0.895, P <0.0001). **(D)** ROC curve of the combination of FNAC and FNA-Tg (AUG: 0.960, P <0.0001). **(E)** ROC curve of the combination of FNAC and the ratio of FNA-Tg to Tg (AUG: 0.962, P <0.0001).

To investigate the optimal cutoff value for diagnosing metastatic cervical lymph nodes in papillary thyroid carcinoma (PTC), we performed receiver operating characteristic (ROC) analysis using postoperative histopathological results (malignant vs. benign) as the state variable, and FNAC, FNA-Tg, the FNA-Tg/sTg ratio, and their combinations as test variables. The optimal cutoff values determined by ROC curve analysis were 32.99 ng/mL for FNA-Tg and 1.55 for the FNA-Tg/sTg ratio. Based on these cutoff values, the area under the curve (AUC) values for diagnosing cervical lymph node metastasis were 0.842 (95% CI: 0.798–0.885) for FNAC, 0.937 (95% CI: 0.907–0.966) for FNA-Tg, and 0.895 (95% CI: 0.853–0.937) for the FNA-Tg/sTg ratio. Furthermore, the AUC for the combination of FNAC and FNA-Tg was 0.960 (95% CI: 0.939–0.982), and the AUC for the combination of FNAC and the FNA-Tg/sTg ratio was 0.962 (95% CI: 0.941–0.983) (**Figure 4**).

### 3.4 Diagnostic evaluation of relevant targets in identifying metastatic neck levels

In our study, when FNAC was used alone as the criterion for evaluating cervical lymph node metastasis, 150 lymph nodes were diagnosed as positive and 169 as negative. Based on final postoperative pathology, three of the positive diagnoses were false positives, and 60 of the negative diagnoses were false negatives. The diagnostic sensitivity, specificity, positive predictive value (PPV), negative predictive value (NPV), and diagnostic accuracy were 71.0%, 97.3%, 98.0%, 64.4%, and 80.3%, respectively.

FNA-Tg is often used as a supplementary diagnostic tool for the clinical evaluation of cervical lymph node status. In our study, the diagnostic sensitivity, specificity, positive predictive value (PPV), negative predictive value (NPV), and diagnostic accuracy of FNA-Tg were 93.7%, 91.0%, 95.4%, 90.3%, and 93.0%, respectively. Given that our previous correlation analysis revealed that serum Tg had a weak but significant influence on FNA-Tg levels, we introduced the FNA-Tg/sTg ratio as a novel diagnostic indicator. For this ratio, the diagnostic sensitivity, specificity, PPV, NPV, and accuracy were 94.2%, 83.0%, 91.5%, 91.0%, and 91.4%, respectively.

**Figure 5:**
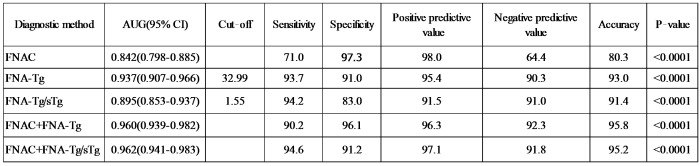
Diagnostic efficiency of relevant methods for diagnosing cervical lymph nodes in PTC patients.

When FNAC was combined with FNA-Tg at the cutoff of 32.99 ng/mL, the diagnostic sensitivity, specificity, positive predictive value, negative predictive value, and accuracy were 90.2%, 96.1%, 96.3%, 92.3%, and 95.8%, respectively. When FNAC was combined with the FNA-Tg/sTg ratio at the cutoff of 1.55, the corresponding values were 94.6%, 91.2%, 97.1%, 91.8%, and 95.2%, respectively (**Figure 5**).

## 4 Discussion

PTC is a malignant tumor originating from thyroid follicular epithelial cells, accounting for over 80% of all thyroid cancer cases. Its most prominent feature is the early occurrence of cervical lymph node metastasis, which is also a significant factor affecting the prognosis and quality of life of patients with PTC. Preoperative assessment of cervical lymph node metastasis directly determines the surgical approach and the extent of lymph node dissection required. Therefore, preoperative evaluation of the benign or malignant status of cervical lymph nodes in patients with PTC is necessary and crucial (19–21).

Numerous methods are currently available for assessing cervical lymph node metastasis. Fine-needle aspiration cytology (FNAC) is the most commonly used preoperative diagnostic method for this purpose (22, 23). However, in clinical practice, factors such as insufficient cellularity in the aspirate, cystic degeneration and necrosis of lymph nodes, and lymph node micrometastasis may occur due to a lack of practical experience and suboptimal operational techniques among ultrasound practitioners. These errors may have a significant impact on subsequent treatment and prognosis (24). In our study, the diagnostic sensitivity, specificity, positive predictive value, negative predictive value, and diagnostic accuracy of FNAC were 71.0%, 97.3%, 98.0%, 64.4%, and 80.3%, respectively. The analysis suggests that the relatively low sensitivity and positive predictive value of FNAC are likely attributable to unavoidable false-negative samples or differences in the structural properties of the lymph nodes. Therefore, it can be concluded that the missed diagnosis rate of FNAC as a standalone method is relatively high, making it unsuitable for screening the benign or malignant status of lymph nodes. However, the misdiagnosis rate of FNAC is extremely low, rendering it highly reliable for diagnosing cervical lymph node status.

Serum thyroglobulin (Tg) is a specific molecule produced by well-differentiated thyroid follicular cells and is involved in the synthesis of thyroid hormones as well as related immune regulation. Small amounts of Tg can be detected in the serum of healthy individuals. However, when thyroid tissue is stimulated or damaged by inflammation, needle aspiration, or increased tumor burden, serum Tg levels typically increase (25). On the one hand, Tg can serve as an objective indicator of tumor recurrence following total thyroidectomy. On the other hand, if Tg is abnormally elevated in non-thyroid tissues (such as lymph nodes), it can provide important evidence of papillary thyroid carcinoma metastasis (26, 27). Therefore, quantitative detection of Tg in lymph node aspirates can serve as one of the key criteria for diagnosing cervical lymph node metastasis.

In 1992, Pacini et al. first proposed that a significant increase in FNA-Tg levels was strongly correlated with cervical lymph node metastasis (28), and subsequent studies confirmed that FNA-Tg had high specificity and sensitivity for diagnosing cervical lymph nodes (29). Therefore, FNA-Tg measurement can serve as a highly reliable tool and an effective complement to FNAC, enhancing the diagnostic accuracy for suspected cervical lymph nodes (30). In this study, we selected a large number of eligible lymph node samples for retrospective analysis to evaluate the role of FNA-Tg. Compared with serum Tg, which showed no significant difference between groups, the median FNA-Tg level in the metastatic lymph node group was significantly higher than that in the non-metastatic group (351.35 ng/mL vs. 0.57 ng/mL). Furthermore, based on FNAC alone, FNA-Tg detection significantly improved the diagnostic accuracy for metastatic lymph nodes, thereby effectively reducing missed diagnoses of some lymph nodes. FNA-Tg detection also helped to exclude certain non-metastatic lymph nodes. In fact, FNA-Tg measurement increased the sensitivity and overall diagnostic performance of FNAC in diagnosing cervical lymph node metastasis in patients with PTC. This strongly suggests that FNA-Tg effectively supplements FNAC in the diagnostic process. This result is consistent with most previous research findings (31, 32).

For a long time, the primary controversy surrounding FNA-Tg has been the lack of a standardized cutoff value. Significant variations are often observed in cutoff values across different centers (33). In previous studies, the wide variation in cutoff values may be attributed to factors such as patient population heterogeneity and differences in techniques or methods. Therefore, in the present study, after minimizing potential confounding factors as much as possible, the optimal cutoff value of FNA-Tg for diagnosing cervical lymph node malignancy, as determined by ROC analysis, was 32.99, with a corresponding AUC of 0.937, indicating high diagnostic value. This finding is broadly consistent with previous research results.

Numerous factors may influence FNA-Tg levels, the most important of which include the state of the thyroid gland itself, as well as serum Tg, TSH, and TgAb. To test this hypothesis, we first compared serum Tg, TSH, and TgAb levels between benign and malignant cervical lymph nodes and found no statistically significant differences, indicating that these markers do not substantially influence cervical lymph node metastasis. Further investigation using Spearman correlation analysis revealed that serum Tg has a moderate positive correlation with FNA-Tg, which may influence the optimal cutoff value and diagnostic accuracy of FNA-Tg for cervical lymph node assessment. Accordingly, based on these statistical findings, we introduced an novel concept—the ratio of FNA-Tg to serum Tg—to evaluate its utility in determining whether cervical lymph nodes have metastasized. In the present study, the FNA-Tg/serum Tg ratio was significantly higher in the metastatic lymph node group than in the non-metastatic group, and both its sensitivity and diagnostic accuracy exceeded those of FNAC alone, making it a useful adjunct for diagnosing malignant cervical lymph nodes. However, similar to FNA-Tg alone, the specificity of the ratio was significantly lower than that of FNAC alone, suggesting that some false-positive cases may still exist in the evaluation process. In summary, to optimize both the sensitivity and specificity of cervical lymph node diagnosis, it is advisable to use FNAC in combination with either the optimal FNA-Tg cutoff value or the FNA-Tg/serum Tg ratio. The diagnostic efficacy of this combined approach is further enhanced compared with the use of either method alone, and the improvement is statistically significant, as demonstrated by ROC analysis. Therefore, integrating these two detection methods in clinical practice is recommended to achieve optimal diagnostic performance.

Finally, based on the above analysis, a preliminary conclusion can be drawn. When FNAC clearly indicates cervical lymph node metastasis, thorough neck lymph node dissection is required in subsequent surgery. When FNAC shows no signs of metastasis, vigilance remains necessary, and FNA-Tg examination should be performed to verify the initial findings. When FNAC indicates no metastasis but FNA-Tg or the FNA-Tg/serum Tg ratio exceeds the optimal cutoff value, we recommend performing cervical lymph node modified radical surgery in addition to radical thyroid cancer surgery to ensure surgical efficacy. This approach can, on the one hand, reduce the risk of postoperative thyroid cancer recurrence and the need for secondary surgery. On the other hand, it can provide substantial support for postoperative pathological staging, potential endocrine therapy or radioiodine therapy, and prognostic follow-up.

The present study has several strengths. First, by utilizing a large dataset in a retrospective analysis, it provides robust evidence for the distinct roles of FNAC and FNA-Tg in diagnosing cervical lymph node status. Second, the novelty of this study lies in the fact that previous research has seldom considered the influence of serum Tg on FNA-Tg. We introduced the ratio of FNA-Tg to serum Tg, which reduces the influence of serum Tg and demonstrates its significant utility in the evaluation of cervical lymph nodes. Nevertheless, several limitations of this study warrant further improvement. First, although the sample size is not small, it is derived from a single center and lacks validation through multicenter clinical trials with unified standards and a consistent patient cohort. Second, due to the short follow-up period, the diagnosis of benign lymph nodes based on FNAC and FNA-Tg testing cannot fully confirm their true benign nature, and there remains a potential for false-negative results. Third, thyroid autoimmune diseases, which often coexist with thyroid cancer, may exert some influence on the measurement of serum Tg or FNA-Tg levels. Therefore, future research should further investigate whether a potential association exists between thyroid autoimmune antibodies and cervical lymph node metastasis. Finally, the lack of long-term follow-up data precludes a full evaluation of the impact of combined diagnostic methods on patient prognosis. Hence, future studies should incorporate long-term follow-up data to assess the prognostic significance and long-term outcomes of these combined diagnostic approaches.

## 5 Conclusion

In summary, ultrasound-guided FNAC remains a gold standard for diagnosing cervical lymph node metastasis in papillary thyroid carcinoma (PTC). However, it has certain limitations. FNA-Tg detection is an objective diagnostic method, and the ratio of FNA-Tg to serum Tg helps reduce the influence of certain confounding factors. Both approaches can significantly improve diagnostic accuracy and demonstrate high diagnostic efficacy. Therefore, they can serve as useful complements to FNAC in the evaluation of cervical lymph nodes. In clinical practice, combining these two markers with FNAC can further enhance the diagnostic value for cervical lymph node metastasis in patients with PTC. A higher positive rate in preoperative cervical lymph node diagnosis not only provides a reliable basis for determining the surgical approach but also plays an important role in disease staging and prognostic follow-up.

## Data Availability

The raw data supporting the conclusions of this article will be made available by the authors, without undue reservation.
